# Prevalence of Color Blindness in Undergraduates of Kathmandu University

**DOI:** 10.31729/jnma.3913

**Published:** 2018-12-31

**Authors:** Reena Kumari Jha, Sukrity Khadka, Yubina Gautam, Manisha Bade, Mukesh Kumar Jha, Ojashwi Nepal

**Affiliations:** 1 Department of Physiology, Kathmandu University School of Medical Sciences, Dhulikhel, Kavre, Nepal; 2 Human Biology, Kathmandu University, Dhulikhel, Kavre, Nepal

**Keywords:** *color blind*, *ishihara plates*, *prevalence*

## Abstract

**Introduction:**

Color blindness is X-linked recessive inherited disorder that occurs mostly in males and is transmitted through females. Many people with color blindness may remain undetected. Thus the present study aims to evaluate the incidence of color blindness among undergraduates of Kathmandu University.

**Methods:**

A cross-sectional study was conducted among 825 undergraduates, aged 17–25 years, from June to August 2018, in Kathmandu University, Kavre, Nepal. The Ishihara plates were used to evaluate the color vision of students under natural day light condition.

**Results:**

Study revealed that 24 (2.9%) undergraduates were color blind which include 24 male (5%) and no female. Among the color blind, five (20.3%), three (12.5%), two (8.33%) and 14 (58.33%) males were the victims of deuteranomaly, deuteranopia, protanomalia and total color blindness respectively. Color blindness is prevalent among the Brahmin 10 (3.9%), followed by Chettri 10 (2.72%) and Newar 4 (2.24%).

**Conclusions:**

Prevalence of color blindness is found to be higher in males than females. Total color blindness is the most prevalent in our study. Screening enables the students to become aware of limitations and devise ways of overcoming them.

## INTRODUCTION

Color blindness, also known as color vision deficiency, is characterized by the inability to perceive different colors clearly. This is an inherited problem; occur due to defect of one or more of the three sets of cones in the eye. Males are more likely to be affected than females.^1^ Human color vision is normally trichromatic that is the mixture of red, green and blue lights. Red-green color blindness is the most common type.^2^ It affects 8% male and 0.5% female worldwide.^3^

Many people with color blind remain undetected either they adapt to the environment or unaware of the disease. They are unfit for certain jobs like traffic policemen, electrician, electronic engineer, doctors which require proper perception of colors.^4^ Early detection allow them to make appropriate decision.

Thus the objective of the present study was to find out the prevalence rate of color blindness in undergraduates of Kathmandu University.

## METHODS

A descriptive cross-sectional study was carried among undergraduates, male and female, aged between 17 to 25 years, at Kathmandu University, Dhulikhel, Kavre. Recruitment was started from June 2018 onwards after taking approval from institutional review committee of Kathmandu University School of Medical Sciences/ Dhulikhel Hospital (IRC-KUSMS). The sample size was calculated by using the following formula:


$$\text{n =}\frac{{(\text{Za/2)}}^{\underline{2}}{}^{*}}{}\frac{\text{P (1-P)}}{{(\text{e)}}^{2}}$$

Where, Za/2 is a normal variant at P< 0.01 having the value of 2.58; P= expected proportion in population based on previous studies (9%)^5^; e= absolute error on precision: 0.025. Thus,


$$\begin{array}{l} \text{n =}\frac{{\left(2.58\right)}^{\underline{2}}}{}\frac{*0.09(1-0.09)}{{\left(0.025\right)}^{2}}\\ \text{n}=825 \end{array}$$

Convenience sampling was done and data collected with the help of Ishihara chart. This chart consists of polychromatic plates containing printed figures made up of colored spots on a background of similarly shaped colored spots.^1^ The figures are intentionally made up of colors that are liable to look the same as the background to an individual who is color deficit. Each participant was asked to sit in adequately lit room and to read all the colored plates of Ishihara chart. The chart was held 75 cm away from the subjects.

The data collected was entered in SPSS (Statistical Package for Social Sciences) version 21 for descriptive analysis. Prevalence of color blindness was calculated by using the following formula.


$$\text{Prevalence}=\frac{\begin{array}{c} \text{Number of all current cases of a}\\ \text{specific disease existing at a given}\\ \text{point in a time x}100 \end{array}}{\begin{array}{c} \text{Estimated population at the same}\\ \text{point in a time} \end{array}}$$

## RESULTS

In the present study, 825 undergraduates [Male 480 (58%), Female 345 (42%); age 17 to 25 years], from different streams of Kathmandu University, Nepal, was assessed for color blindness (Table 1).

**Table 1:** Anthropometric variable

Gender	Male	Female	Total
n (%)	480 (58.2%)	345 (41.8%)	825

In Overall population, 24 were color blind with a prevalence of 2.9%. Among 480 boys, 24 were found to have color blindness with prevalence of 5% however none of the girls were victim (Table 2).

**Table 2:** Prevalence rate of color blindness.

	Male	Female	Total
No. of Students Examined	480	345	825
No. of Color Blind Students n (%)	24 (5)	0 (0)	24 (2.9)

The distribution of different types of color blindness among subjects of the present study is presented (Table 3).

**Table 3:** Different types of color blindness and their prevalence rate.

	Deuteranomaly		Deuteranopia		Protanomalia		Total CVD	
Gender	Male	Female	Male	Female	Male	Female	Male	Female
n (%)	5 (20.83)	0 (0)	3 (12.5)	0 (0)	2 (8.33)	0 (0)	14 (58.33)	0 (0)

Among the color blinds, 5, 3, 2 and 14 males were the victims of deuteranomaly, deuteranopia, protanomalia and total color blind respectively. Table 4 shows color blindness is prevalent among the Brahmin (3.9%), followed by Chettri (2.72%) and Newar (2.24%).

**Table 4:** Distribution of color blindness of the subjects according to Ethnic groups.

Ethnicity	Subject Examined			Subjects with Color Blind			Prevalence Rate
	Male	Female	Total	Male	Female	Total	
Brahmin	165	92	257	10	0	10	3.89%
Chettri	215	152	367	10	0	10	2.72%
Newar	92	86	178	4	0	4	2.24%
Rai/Gurung/Tamang/Dunuwar/Rajbansi	14	9	23	0	0	0	0%

## DISCUSSION

In our findings, the prevalence of color blindness among the boys were found to be similar recorded by Pramanik 5.58%,^6^ Kharel 5.12%,^7^ Masood 6.36%.^3^ However the prevalence of the color blindness (male) in our present study is higher than that of Shrestha 3.9%,^8^ Niraula 3.8 %,^9^ 2.9% in Arabian children,^10^ Libya 2.2%,^11^ India 2.3%. ^11^ Color blindness is an inherited problem thus the genes responsible for the most common forms of color blindness are located on the X chromosome. As females have two X chromosomes, a defect in one is typically compensated by the other, while males have only one X chromosome. They are commonly suffered. ^1^ None of the girls in our study were found to be color blind, which corroborates with the some researches done in Nepal,^8,9^ India,^12^ Spain,^13^ but in few studies color blindness were detected among girls, 0.40%,^14^ 0.84%,^3^ 2.56%.^7^ This higher occurrence of the condition in males suggests that the prevalence of CVD in the population is gender specific.

Out of 24 color deficits, 5 (20.83%) were found to have deuteranomaly, 3 (12.5%) with deuteranopia, 2 (8.33%) with protanopia and 14 (58.33%) had total color vision deficiency. the prevalence rate of deuteranopia was higher (50%), deuteromaly (33%), and Protanomaly (17%). In contradictory to our study, none of them were found to have total color defecit.^9^

The frequency of red/green color blindness was found to varying between different races, tribes and ethnic groups. In this study color blindness is more prevalent in Brahmin and Chettri followed by Newar. Godar^5^ found color blind is highest in Chettri followed by Brahmin and Newar however Niraula^9^ found the highest prevalence in Darji followed by Newar.

## CONCLUSIONS

Higher occurrence of the color blindness in male suggests that the prevalence of color blindness in the population is gender specific. Since the prevalence is found highest among undergraduates, it is recommended to screen all students before they enroll in specific field. If this disorder can be detected at earlier and proper counseling is done, regarding the limitation, a large number of people can be saved from the psychological stress of having chosen wrong field.
